# Supplementary material for: Isolation and Investigation of Potential Non-Saccharomyces Yeasts to Improve the Volatile Terpene Compounds in Korean Muscat Bailey A Wine
Source: Microorganisms. 2020 Oct 8;8(10):1552. doi: 10.3390/microorganisms8101552 (PMC7601120; doi:10.3390/microorganisms8101552)
Supplement: Supplementary file 1 [file microorganisms-08-01552-s001.pdf]

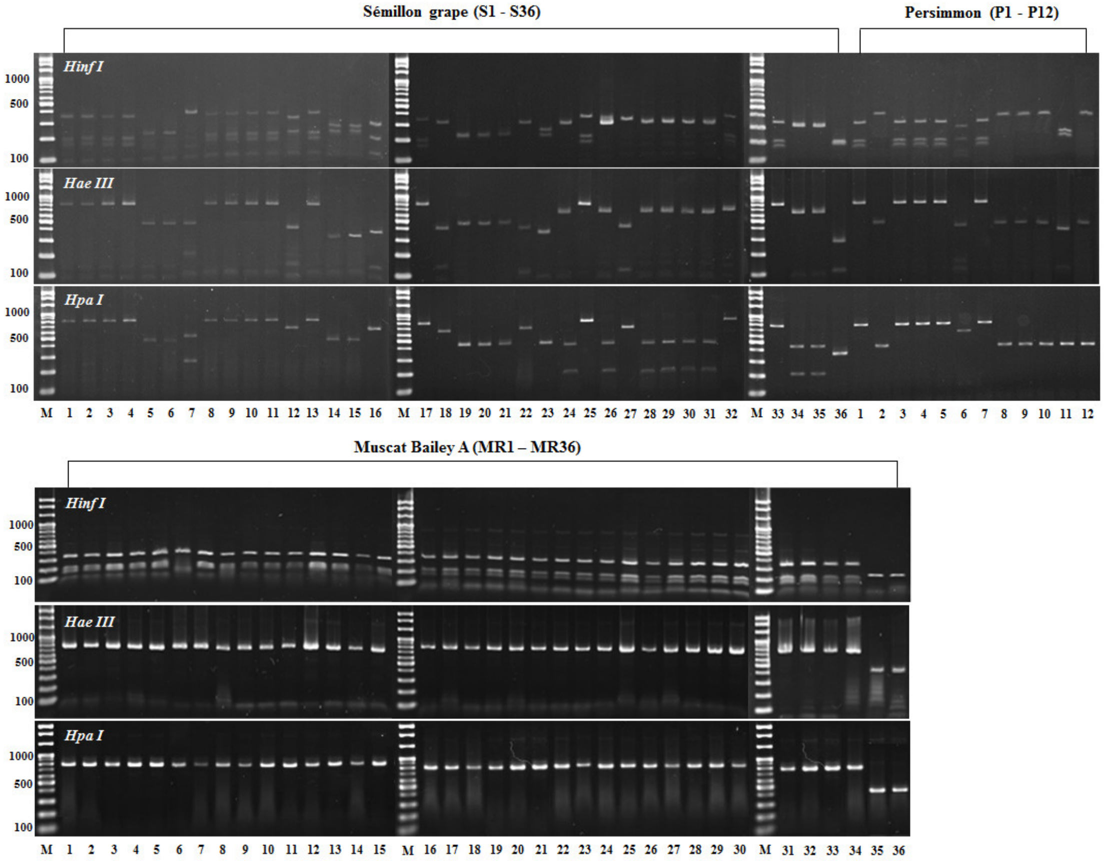

Figure S1. Polymerase chain reaction restriction fragment length polymorphism (PCR-RFLP) analysis of amplified ITS I-5.8S-ITS II region of 84 isolates from Sémillon grape (S), persimmon (P), and Muscat Bailey A grape (MR). The DNA fragments were amplified from the yeast chromosomal DNA with ITS1 and ITS 4 primers, digested with *Hinf* I, *Hae* III, and *Hpa* I, and resolved on a 2.0% agarose gel. Lane M represents a 100-bp plus DNA ladder used as a DNA size marker.

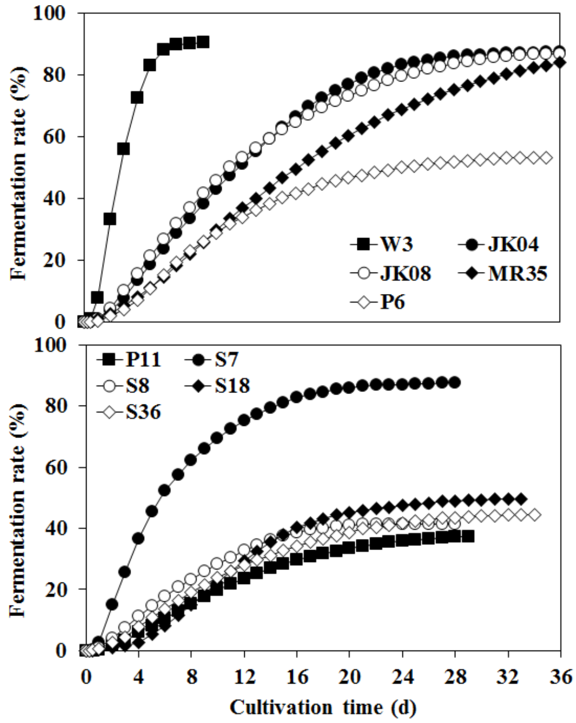

Figure S2. Fermentation rates of nine non-*Saccharomyces* yeasts and the control strain *Saccharomyces cerevisiae* W-3. Fermentation rate was calculated based on carbon dioxide reduction from the total glucose concentration (20%) until the completion of ethanol fermentation. W3, *S. cerevisiae* W-3; JK04, *Wickerhamomyces anomalus* JK04; JK08, *Torulaspora delbrueckii* JK08; MR35, *Starmerella bacillaris* MR35; P6, *Candida quercitrusa* P6; P11, *Pichia kluyveri* P11; S7, *Hanseniaspora vineae* S7; S8, *H. uvarum* S8; S18, *C. railenensis* S18; S36, *Metschnikowia pulcherrima*.

Table S1. Diversity of yeasts isolated from various food materials such as Sémillon grape (S), persimmon (P) and Muscat Bailey A grape (MR).

Strains	Number of strains depending on the origin			Isolates
	Sémillon grape	Persimmon	MBA grape	
Hanseniaspora uvarum	13	5	34	S (1-4, 8*-11, 13, 17, 25, 32, 33), P (1, 3-5, 7), MR (1-34)
Starmerella bacillaris	5	5	2	S (5, 6, 19-21), P (2, 8-10, 12), MR (35*, 36)
Candida quercitrusa	1	1		S (16), P (6*)
Pichia kluyveri	3	1		S (14-15, 23), P (11*)
Hanseniaspora vineae	9			S (7*, 24, 26, 28, 29-31, 34, 35)
Candida railenensis	4			S (12, 18*, 22, 27)
Metschnikowia pulcherrima	1			S (36*)
**Total**	36	12	36	84

The isolates marked with an asterisk (*) showed the highest OD value among each of the species when cultured in sterilized MBA grape juice at 30 °C for 48 h and were selected for further study.
